# Supplementary figures and images for: A paralog-specific role of COPI vesicles in the neuronal differentiation of mouse pluripotent cells
Source: Life Sci Alliance. 2020 Jul 14;3(9):e202000714. doi: 10.26508/lsa.202000714 (PMC7368096; doi:10.26508/lsa.202000714)

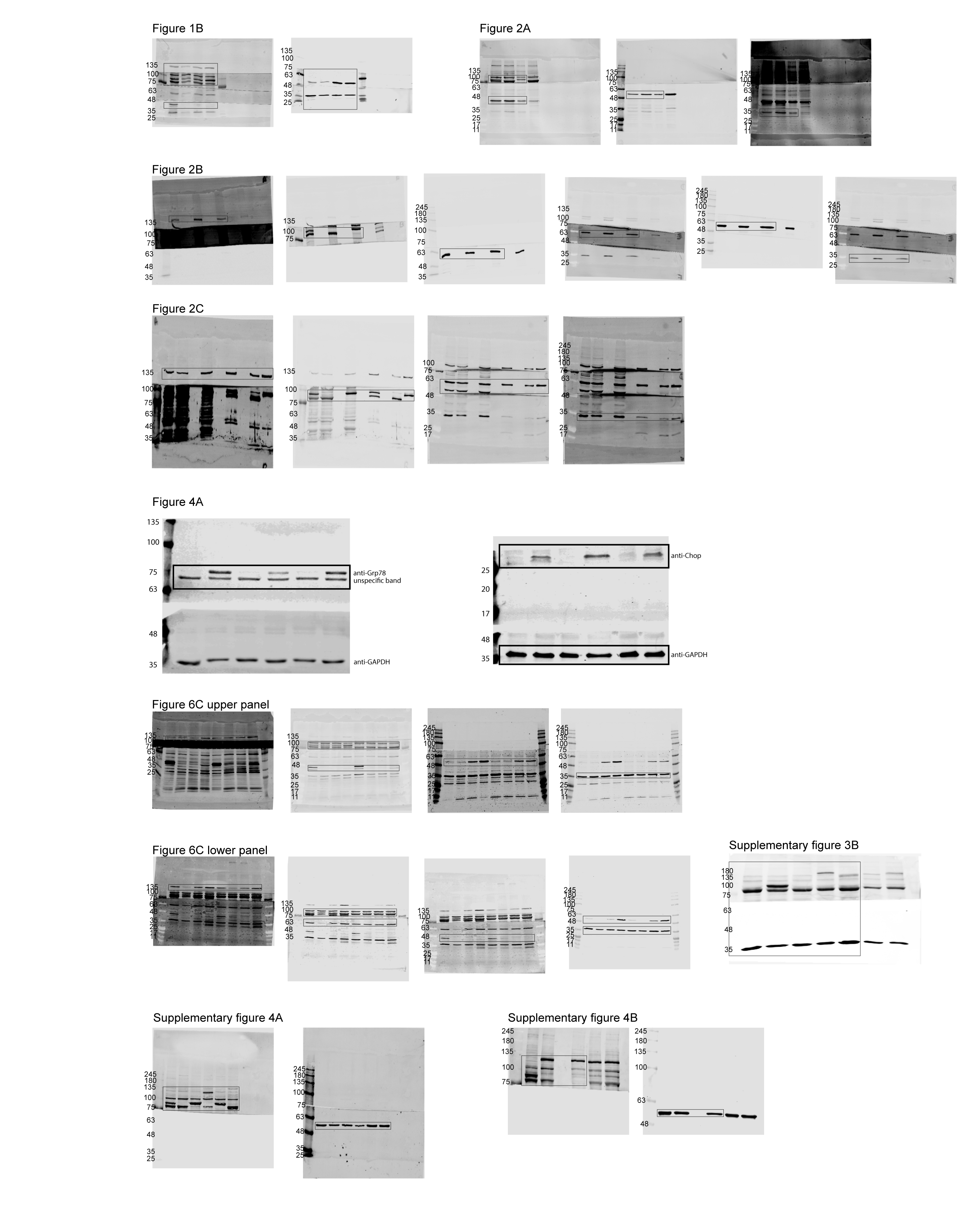

Supplement: Supplementary file 1 [file LSA-2020-00714_SdataF1_F2_F4_F6_FS3_FS4.tif]
